# Human's Capability to Discriminate Spatial Forces at the Big Toe

**DOI:** 10.3389/fnbot.2018.00013

**Published:** 2018-04-10

**Authors:** Annette Hagengruber, Hannes Höppner, Jörn Vogel

**Affiliations:** German Aerospace Center (DLR), Institute of Robotics and Mechatronics, Weßling, Germany

**Keywords:** tactile feedback, haptics, haptic display, teleoperation, prosthesis, human-in-the-loop, sensory substitution

## Abstract

A key factor for reliable object manipulation is the tactile information provided by the skin of our hands. As this sensory information is so essential in our daily life it should also be provided during teleoperation of robotic devices or in the control of myoelectric prostheses. It is well-known that feeding back the tactile information to the user can lead to a more natural and intuitive control of robotic devices. However, in some applications it is difficult to use the hands as natural feedback channels since they may already be overloaded with other tasks or, e.g., in case of hand prostheses not accessible at all. Many alternatives for tactile feedback to the human hand have already been investigated. In particular, one approach shows that humans can integrate uni-directional (normal) force feedback at the toe into their sensorimotor-control loop. Extending this work, we investigate the human's capability to discriminate spatial forces at the bare front side of their toe. A state-of-the-art haptic feedback device was used to apply forces with three different amplitudes—2 N, 5 N, and 8 N—to subjects' right big toes. During the experiments, different force stimuli were presented, i.e., direction of the applied force was changed, such that tangential components occured. In total the four directions up (distal), down (proximal), left (medial), and right (lateral) were tested. The proportion of the tangential force was varied corresponding to a directional change of 5° to 25° with respect to the normal force. Given these force stimuli, the subjects' task was to identify the direction of the force change. We found the amplitude of the force as well as the proportion of tangential forces to have a significant influence on the success rate. Furthermore, the direction right showed a significantly different successrate from all other directions. The stimuli with a force amplitude of 8 N achieved success rates over 89% in all directions. The results of the user study provide evidence that the subjects were able to discriminate spatial forces at their toe within defined force amplitudes and tangential proportion.

## 1. Introduction

Tactile perception is essentially involved in manual dexterity. The interaction of sensory input (tactile feedback) and motor output (movement) at our hands allows for a dexterous manipulation of objects. The sensory information produced by the mechanoreceptors in the glabrous skin of our hands allows for manipulation tasks such as precisely lifting an object in a pinch grip, which has been investigated intensively (Johansson and Flanagan, 2008). The importance of tactile feedback can be seen for example in experiments with anesthetized digits as done in Johansson et al. (1992) and Nowak et al. (2001). With the evoked absence of the tactile grasp information of the digits the regulation of the grip force is impaired leading to a considerably reduced success of grasping. Even if visual feedback is still an essential feedback during grasping, the mechanoreceptors in the hands provide information about shape and stiffness, which can override the visual prediction (Johansson and Flanagan, 2008). Haptic Feedback is nowadays state-of-the-art in teleoperation and in virtual reality. It can be provided by various haptic feedback devices and can be composed of kinesthetic or tactile sensation. Naturally, the haptic sense consists of both, tactile and kinesthetic sensing (Anderson et al., 1999). While the kinesthetic sensing provides perception of limb position and movement from the mechanoreceptors in the muscles, tactile sensation provides touch information and is generated by the mechanoreceptors in the skin (Jones, 2000). Designs of both modalities are possible (e.g., in haptic feedback devices Fritschi et al., 2006; Meli et al., 2014).

Also modern teleoperated robotic systems are able to feed back forces from the task scene. An example for this is the DLR HUG (Hulin et al., 2011). It provides, besides the visual feedback, haptic information of the remote robot directly to the hands of the operator. A more complex system is the DLR MIRO (Hagn et al., 2008), a system for minimal invasive surgery, which consists of three robotic arms equipped with different force and torque sensing technologies. Such complex systems may require additional feedback channels to provide more information to the surgeon. Another robotic systems is the BairClaw (Hellman et al., 2015), a robotic finger equipped with different haptic sensors which can provide remote information to the user.

Due to the importance of the grasp information, the lack of feedback is also a major issue in upper limb prosthesis. Consumer studies on amputees using hand-prosthesis showed that one of the most wanted features they would love to be added is to feel the forces occurring during grasping (Biddiss et al., 2007; Pylatiuk et al., 2007). Plenty of invasive and non-invasive feedback approaches for hand prosthesis were investigated in research. In invasive methods nerves are often directly stimulated by implanted electrodes (e.g., Dhillon and Horch, 2005; Rossini et al., 2010; Raspopovic et al., 2014). A different invasive approach is the Targeted Muscle Reinervation (TMR), where the nerve endings are reinervated in available muscles (Kuiken et al., 2009). Non-invasive techniques include methods of electrical and mechanical (force or vibrotactile) stimulation. Examples thereof can be seen in Patterson and Katz (1992), Cipriani et al. (2008), Witteveen et al. (2012), Meek et al. (1989), and Antfolk (2012). Furthermore, neural interfaces for amputees which provide force feedback are discussed in Hellman et al. (2015). However, tactile feedback is not available in commercial hand prosthesis yet. While most investigations show a positive influence for the control of the prosthesis, the lack of product availability is possibly caused by different reasons. Firstly, in studies of non-invasive methods, the feedback is often applied to the hairy skin of the body (e.g., forearm and upper arm). However, the mechanoreceptors in the hairy skin provide different sensory information as those in the glabrous skin of the hands (Vallbo et al., 1995; Koeppen and Stanton, 2010). Furthermore, the space on the forearm is usually occupied by the shaft of the prosthesis, which makes it troublesome to access this area for feeding back sensory information.

Some of the aforementioned systems show applications where the usage of our hands as natural feedback channel is limited or impossible. Either in prosthesis, where the hand is not available at all, or in teleoperated robotic systems, where the hands and respective feedback channels may already be overloaded with other tasks. New feedback approaches can help to address this problem. One promising approach is to provide the grasp information to the bare front side of the toes. This approach is of special interest, since the neural structure of the skin of the toe shows similarities to that in the hands (Kennedy and Inglis, 2002). All four kinds of mechanoreceptors, which are known to exist in the glabrous skin of the hand, are available in the glabrous skin of the foot as well. A particularly interesting region of the foot sole is the big toe, where three of the four mechanoeceptors found in the hand are also available. This may lead to superior perception capabilities compared to other skin regions. Looking at the two-point discrimination threshold, it is evident, that the value at the toe with 9–10 mm is closer to that of the finger (2–3 mm) as compared to other body parts with hairy skin (35 mm) (Panarese et al., 2009).

In Panarese et al. (2009) uni-directional forces representing the grasp force in a teleoperation task were applied to the subjects' toes. Their work showed that the mechanoreceptors at the toe allow for embedding uni-directional force feedback into the sensorimotor-system, which improved the control of a robotic hand. Furthermore, it demonstrates the basic concept, i.e., that humans are able to close the loop between (artificial) motor functionality provided at the hand and sensory information given to the toe. However, in contrast to the human hands, literature lacks about psychophysical analysis at the toe for spatial force feedback. The perception in hands and fingers is a broad topic with many interesting findings about the perception and discrimination abilities. Among others, these involve investigations of discrimination of curvature (Gordon and Morison, 1982), vibrotactile frequencies (Franzén and Nordmark, 1975), or gratings (Sinclair and Burton, 1991). Furthermore, for the fingers it is known that the mechanoreceptors allow not only discrimination of uni-directional feedback but also the discrimination of spatial forces. Panarese and Edin (2011) showed that the mechanoreceptors of the skin of fingertips enable the discrimination of three-dimensional (spatial) forces. A mechanical force of 5 N was applied to the index fingertip of twelve participants. The authors found that a minimal tangential angle of 7.1° could be perceived. Another study of Wheat et al. (2004) could demonstrate human's ability to discriminate tangential forces at the fingers during grasping. In an additional proof of concept, we were able to show that spatial toe force-feedback can be successfully integrated into the sensorimotor control for teleoperating a robotic arm (Hagengruber et al., 2017). However, literature is lacking comparable fundamental studies of how well humans are able to discriminate spatial toe force-feedback.

Based on these findings, this study aims at analyzing the capability of humans for discriminating spatial forces at the bare front side of the big toe. In order to achieve this, we performed a study with 24 healthy subjects. During the experiments various force stimuli were presented to subjects' toe using a standard force feedback device. In each trial the force vector changes its direction with respect to the force acting normal to the toe. Four directions are used: up (distal), down (proximal), left (medial), and right (lateral). The proportion of the tangential force was varied in each stimulus. It changes between 5° and 25° with respect to the normal acting force. Furthermore, catch-trials, in which no tangential force component was present, were applied. The tests were performed three times with absolute force amplitudes of 2 N, 5 N, and 8 N. A modified state-of-the-art haptic feedback device was used to realize the stimulation of the toe. The experiments present a pure investigation on the perception at the toe and not the integration of a feedback to the sensorimotor-control. The haptic device provided forces to a specific location of the skin and thereby relies more on tactile than on kinesthetic sensation. It is known that such approaches do not impair the perception of the feedback (Meli et al., 2014; Pacchierotti et al., 2014). Relying on tactile sensation may be of importance with respect to creating a miniaturized and wearable toe-feedback-device.

## 2. Methods

In this section we will outline the experimental design, explain used equipment as well as the experimental protocol and will present the statistical model for investigating dependencies between factors and defined metrics.

### 2.1. General description and participant task

Since object manipulation provides normal as well as tangential force information, the experiment is designed to cover both force types. Therefore, two main tests were implemented: a Tangential Test (TT) and a Normal Test (NT). The Tangential Test is designed to investigate the capability for discriminating spatial forces at the toe, meaning the possibility to discriminate for certain directions is of interest. Whereas the Normal Test deals with the participants' perception of changes in the normal-force amplitude. In each test a reference force was applied to the glabrous skin of the distal phalanx of the subjects' right big toe. Depending on the test, either the direction of force (TT) or its amplitude (NT) was changed in each trial with respect to this reference force. Subjects were asked to identify the presented force change at the toe either in amplitude or direction. In total, the subjects were asked to perform four test cycles (one Normal Test and three Tangential Tests). The NT was performed with a reference contact force of 5 N. Starting at this reference, the force was either decreased, or increased and returned back to the reference after each trial. This test allows to draw conclusions about the minimal required change in amplitude to be detected at the toe. The NT was always performed first by the subjects. The TT is designed to investigate if a spatial discrimination at the toe is possible at all. Furthermore, it allows to draw conclusions whether the proportion between tangential and total force amplitude has an influence on this discrimination. The TT was performed at three different Force Levels of 2 N (low), 5 N (medium), and 8 N (high). For each Force Level, the factor Direction (up, down, left, and right) as well as the Tangential Component (5° to 25°) was varied randomly. The participants were asked to enter the perceived force change at their toes via the number pad of a computer keyboard. To guide the subjects through the test, a graphical user interface (GUI) was implemented. The GUI visualizes the possible answers for selection. For the NT *increased, decreased*, or *same* is displayed, whereas for the TT the options *up, down, left, right*, and *same* were available. As soon as the subjects reported their decision on the given stimulus, they could start the next stimulus by pressing the space bar, which allowed them to take as much time as they need for the experiment. Depending on the decision time, a test cycle (NT, or one TT) lasted between 4 to 9 minutes. The subjects had to complete a training phase before the main tests to become familiar with the experimental procedure and the amount of applied force. Only during this training, the subjects got experimenter feedback about correctness of their decision. The results of the training have not been used for the analysis and thus, were not recorded. In order to ensure identical experimental conditions, subjects had to sit — not walk or stand — 5 min before starting the experiments. A 5 min break was included between tests accordingly. During each break a questionnaire had to be filled, in which the subjects were asked about their mental demand, their self-estimation in performance, their frustration level, and their comfort during the test. Each of these metrics had to be rated on a scale of 1–20, with 1 corresponding to very low/very well and 20 to very high/very bad. The NASA TLX questionnaire (Hart and Staveland, 1988) served as a guideline. The average time for the whole test procedure was about 70 min.

### 2.2. Participants

A total of 24 healthy subjects including 20 men and 4 women, age 21–38 years, performed the experimental protocol as described above. No subject had a reported history of neurological disorder or neuromuscular injury affecting the CNS or the muscles. All subjects participated voluntarily and gave written consent to the procedures, which were conducted in partial accordance with the principles of the Helsinki agreement (non-conformity concerns the point B-16 of the 59th World Medical Association Declaration of Helsinki, Seoul, October 2008: no physician supervised the experiments). Approval was received from the works council of the German Aerospace Center, as well as its institutional board for data privacy ASDA; the collection and processing of experimental data were approved by both committees. Before starting the experiments, subjects were quickly briefed by describing them the experimental procedure and the goal of the experiments. The experimental setup was adjusted to each subject such that they felt comfortable and the stimulation could be performed properly.

### 2.3. Experimental setup

**Figure 1:**
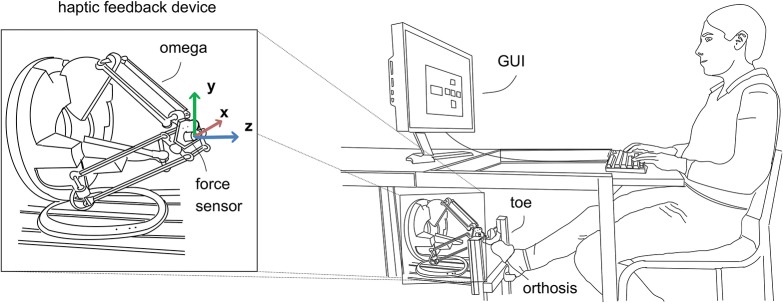
Experimental setup. **(Left)** Haptic feedback device omega.3 with mounted force-torque sensor. The coordinate system is depicted, with its origin at the tip of the device. **(Right)** The whole setup includes the haptic feedback device, a foot orthosis as well as a foot shell to fix the toe in front of the feedback device, and a graphical user interface that allows the subject to provide feedback about the perceived stimulation at the toe. The interface is realized by a screen and a keyboard on a table with adjustable height.

The setup includes the haptic feedback device, an adjustable foot shell with a fixation for the toe, and a table with adjustable height with a screen and a keyboard on top. The setup is depicted in Figure 1. The stimulation was realized with the modified haptic feedback device omega.3 of the company Force Dimension (Force dimension, 2013). The device is a delta-based parallel kinematic with active gravity compensation and 3 Degrees of Freedom (DoF). It provides a cylindrical work space with a diameter of about ∅ 160 mm and length of 110 mm. Furthermore, it allows for a maximum force of 20 N and a stiffness of 14.5 N/mm. To measure the exact interaction forces between toe and device the DLR Fingertip sensor was mounted to the end-effector of the haptic device. The dimensions of the cylindrical 6 DoF force-torque sensor are ∅ 30 × 17 mm. It is based on a strain-gauge technology and designed for forces of up to 30 N in each direction. The sensor allows for a closed control loop to adjust the applied forces at the toe using a PID controller. The control software for the haptic device was developed in MATLAB Simulink and executed on a Linux based real-time computer. Implementation of the user interface and the test protocol was also realized in MATLAB and MATLAB Simulink.

**Figure 2:**
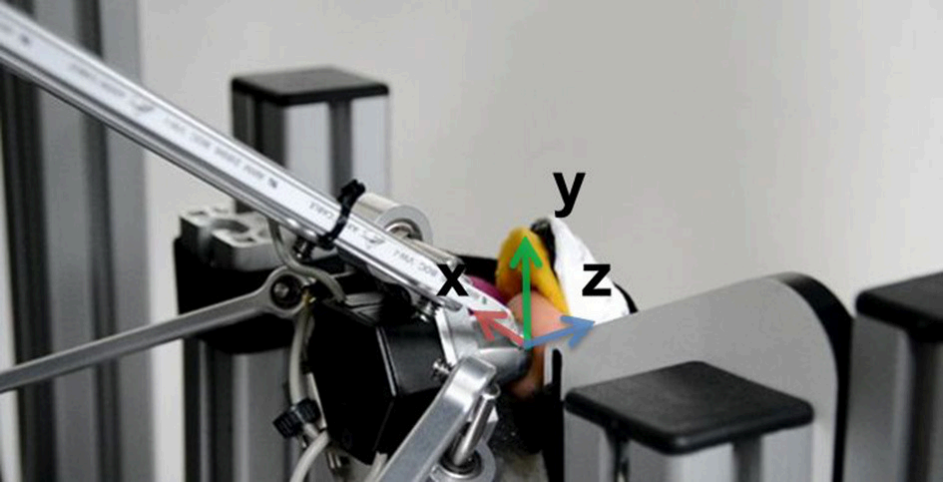
The experimental setup with a close-up view from the toe. The toe is fixed in front of the haptic feedback device. The device stimulates the bare front side of the distal phalanx of the toe.

For an optimal skin connection a hemispheric plastic tip with comparably high stiffness (in relation to the stiffness of human skin) having a diameter of 10 mm was mounted at the force sensor. The modified haptic device allowed the stimulation of spatial forces of up to 10 N at a maximum frequency of 5 Hz. The device is mounted out of view for the subjects, i.e., below the table and 200 mm above the ground, in order to prevent subjects being influenced by visual feedback of the devices movement. Additionally, the participants were equipped with hearing protection to block any acoustic information originating from the feedback device and to avoid distraction due to surrounding sounds. An adjustable foot shell in front of the feedback device holds the foot in the right position. The ball of the foot props to a wooden plate. A slight angle occurs at the joint between the first proximal phalanx and the metatarsal bone. The toe is straightened and the glabrous skin of the toe is positioned perpendicular to the haptic device. A stabilizing orthosis made of medical grade thermoplastic is used to immobilize the toe. It guarantees a rather fixed stiffness of the toe's joints during the tests. Without the orthosis, the applied force could have been compensated by the subjects. A close-up view of the toe and the haptic device can be seen in Figure 2. The z-axis acts perpendicular to the skin of the toe, the y-axis acts to the distal, and the x-axis to the lateral side of the toe, respectively.

### 2.4. Force pattern

**Figure 3:**
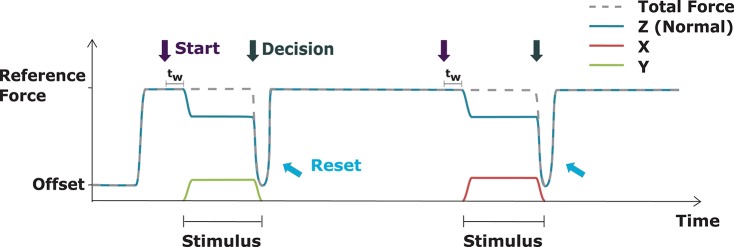
Schematic illustration of the Tangential Test. The blue line indicates the z-component, which acts plantar to the toe, red the x-component which acts medial-lateral, and green indicates the y-component which acts proximal-distal. The pure z-component represents the reference for the tangential stimulation. Different directional stimuli occur and are preserved until the decision for a direction has been taken. When the stimulus is started a randomly selected waiting time *t*_*w*_ is applied. The trial is terminated by resetting the force to the starting point.

The Tangential Test is based on changes of the effective direction of the force. A schematic illustration of the force pattern is given in Figure 3. In order to stabilize the contact of the stimulation device to the skin, an offset force of 0.5 N is initially applied, as soon as the subject is in position. Once the subject starts the test cycle the normal force is increased to the respective Force Level of the test (2 N, 5 N, or 8 N). This pure normal force (plantar to the toe, i.e., in z-direction) represents the reference force to which the subject compares the stimulus. The individual trials of the tests were started by pressing the space bar. After a randomly selected waiting time *t*_*w*_ (1*s* ≤ *t*_*w*_ ≤ 1.5*s*), the actual stimulus is applied. In the TT, the total force of the stimulus is being kept constant. Consequently, the presence of the tangential force (±x or ±y) results in a decrease of normal force. The exact values of the tangential and normal parts can be seen in Table 1. The portion of the Tangential Component was selected from the discrete levels of 0°, 5°, 10°, 15°, 20°, or 25°. To achieve a smooth transition from reference force to the actual stimulus, the application of the stimulus is blended with a scaled 2 Hz half-sinusoid waveform. This blending function was selected analogously to the work by Panarese et al. (2009). After the directional stimulus is fully achieved, it stays constant until the subject has given its decision about the perceived Direction of stimulation. Upon decision, the force is reset to the reference within the same time as the Tangential Component returns to zero. During this process the Normal Component decreases shortly to the offset of 0.5 N and then back to the reference force. This way, no further haptic information about the previous stimulus is provided to the subject. After resetting, the reference force is constantly applied until the next trial is initiated by the subject pressing the space bar.

**Table 1:** Exact values for the Normal Component (acting in z-direction, plantar to the toe) and the Tangential Component (+x: lateral; −x: medial; +y: distal; −y: proximal) for the displacements of 0°, 5°, 10°, 15°, 20°, and 25°.

** 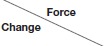 **	**Low [N]**		**Medium [N]**		**High [N]**	
	**Normal**	**Tangential**	**Normal**	**Tangential**	**Normal**	**Tangential**
0° (same)	2	0	5	0	8	0
5°	1.99	0.17	4.98	0.44	7.97	0.70
10°	1.97	0.35	4.92	0.87	7.88	1.39
15°	1.93	0.52	4.83	1.29	7.73	2.07
20°	1.88	0.68	4.70	1.71	7.52	2.74
25°	1.81	0.85	4.53	2.11	7.25	3.38

*The acting force vector results from both components*.

Each TT comprises five stimuli in four directions and four catch trials. This sums up to 24 different stimuli, which were randomized and repeated three times each. This 72 stimuli were repeated for each of the single Force Levels (i.e., 216 stimuli in total). In order to detect whether the order of presentation of the three Force Levels makes any difference, we permuted it resulting in six different constellations (3!). With a total of 24 subjects, each possible constellation was performed by four subjects.

The force pattern of the Normal Test follows the same scheme as the Tangential Test. The initial force of 5 N represents the reference force. Starting at this level, the stimulus consists either of an increased or decreased normal force. No tangential forces are applied here. As prior tests indicated that an increase in force is easier to detect than a decrease in force, more stimuli of decreasing forces opposed to increasing force were used. Therefore, increasing stimuli occur in the range of +0.25 N to +2.0 N sampled at 0.25 N steps. Whereas, decreasing stimuli occur from −0.25 N to −2.75 N with −0.25 N steps. This sums up to a total of 20 different stimuli with increasing or decreasing force. Similar to the TT, a reset sequence occurs after each decision and before the reference force is applied again. The different stimuli were repeated three times each, summing up to a total of 60 stimuli for the NT. The randomization has been performed within the 20 different trials.

### 2.5. Data analysis

For statistical analysis a logistic regression model with fixed and random effects based on Equation (1) is used. The fixed values are given by the vector of parameters $\beta^{T}=\left(\beta_{0},\beta_{1},\ldots ,\beta_{m}\right)$ and the vector of influential variables **x**. The random effect is given by γ and the error term by ε.

(1) $$\begin{array}{r} log\left(\frac{P}{1-P}\right)={\text{x}}^{T}*\beta +\varepsilon +\gamma \end{array}$$

Here, the vector of parameters is defined by the factors Level and Direction, whereas the only influential variable is the factor Degree. For the analysis of NT the factor of Direction (increase – decrease) is the fixed parameter and the Force Change acts as the influential variable. The correct answer per trial with *y*_*i*_ ∈ {0, 1} is used for the analysis. The direction *same* was not considered in the deeper analysis. These catch trials were recognized with close to 100% and showed no further information about the directional recognition. The statistical analysis was performed in R. Based on the logistic regression model the Just Noticeable Difference (JND) averaged over all subjects can be determined. This psychometric value relates to the difference required in a stimulus, such that the subjects are able to notice it on 50% of the trials. This recognition-rate is clearly higher than the chance-level of 20% (one out of five possible answers).

## 3. Results

In the following, the results for the Tangential and the Normal Test and their statistical analysis are presented.

### 3.1. Tangential test

The collected data include the correct answers with *y*_*i*_ ∈ {0, 1}, each assigned to a factor Force Level (low, medium, and high), Direction (up, right, down, and left), and Degree of Tangential Component (0°, 5°, 10°, 15°, 20°, and 25°). Table 2 shows the estimated values of the logistic regression model. The shown results relate to the used base (i.e., level *low* and direction *down*). The values in *estimate coefficient* describe the increase of the odds for a correct answer in comparison to the base value. Since the analysis is based on a logit-model, it originally provides results in a logarithmic scale. For better readability, the values for *estimate coefficient* are converted to linear scale. Thus, for example, the odds for a correct answer on a trial at level medium in comparison to the base low is increased by a factor of *exp*(β_*Low*_) = 4.08. The *p*-values with the corresponding std. error in the table show again the influence to the odds. The used logit-model is based on the two factors Degree and Level, which showed significant interaction.

**Table 2:** Estimated value of the logistic regression model.

**Parameters**	**Estimate coefficient**	**Std. error**	***p*-value**
β_0_	0.02	0.35	<2e-16
β_*Level, Low*_	1	−	−
β_*Level, Medium*_	4.08	0.27	2.45e-7
β_*Level, High*_	6.47	0.26	1.30e-11
β_*Direction, down*_	1	−	−
β_*Direction, up*_	0.94	0.12	0.60
β_*Direction, left*_	0.87	0.12	0.24
β_*Direction, right*_	0.65	0.12	2.4e-4
*Degree*	1.22	0.02	<2e-16
*Degree* : β_*Level, Medium*_	1.03	0.02	0.09
*Degree* : β_*Level, High*_	1.06	0.02	1.0e-3

*Given are all values of the parameter vector **β** and the influential variable degree of the logistic regression model. Parameters linked with “:” indicate the interaction of these factors*.

The statistical analysis shows that all factors have a significant influence on the directional discrimination. The interaction of the factors Force Level and Degree of Tangential Component is significant as well. This implies that the Tangential Component influences the results dependent on the applied total force and vice versa. Furthermore, it can be seen that the direction right was recognized significantly worse than the other directions. The factor Direction shows no significant interaction with the other factors.

**Figure 4:**
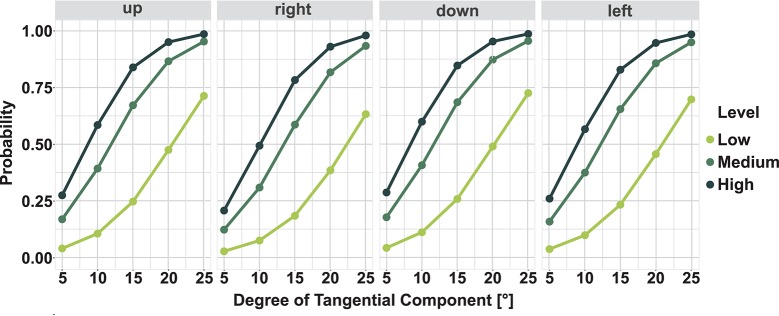
Probability to recognize a tangential force stimulus per Levels and Direction. The curves give the probability from 0 to 1 to successfully recognize a tangential force stimulus per Level and Direction. The Just Noticeable Difference (JND) can be read at 50% probability.

At the lowest level of 2 N a maximum of about 60% of correct answers is achieved. The medium level (5 N) shows maximum results of about 80%, and the highest level with 8 N reaches correct answers up to 90% and above. The direction *same* achieved a considerably higher recognition-rate of 92% and above in all levels. The used regression model allowed to calculate the probability to recognize a tangential force stimulus depending on the given factors. Figure 4 shows this probability per Level and Direction. The maximal tangential stimulus of 25° leads to a probability close to one in medium and high level. However, at the low level only a probability of 0.75 is achieved. This means, that the probability to recognize a tangential stimulus in the high level is much higher than for a stimulus in the low Force Level. Finally, the 50% recognition-rate can be determined which indicates clear differences in the three Force Levels. Table 3 lists the values for the 50%-JND per Level and Direction. In the level low more than 20° of Tangential Components were necessary to achieve a recognition-rate of 50%. The JND of the medium level is between 11.6° and 13.5° depending on the direction. At the high level, the estimated JND is between 8.4° and 10.1°.

**Table 3:** Just Noticeable Difference (JND) per Level and Direction.

**Level**	**Direction**	**JND [°]**
Low	Up	20.5
	Right	22.3
	Down	20.2
	Left	20.8
Medium	Up	11.9
	Right	13.5
	Down	11.6
	Left	12.2
High	Up	8.7
	Right	10.1
	Down	8.4
	Left	9.0

*The JND is the difference that subjects are able to notice in 50% of trials. The values show that the JND changes for the different Force Levels*.

At the Force Level of 2 N only the 25° stimulus is larger than the JND at this level. In this stimulus a tangential force of 0.85 N (and 1.81 N in z) is applied to the toe. The exact values can be taken from Table 1. A stimulus corresponding to the JND at this level would have a Tangential Component of 0.72 N considering the average JND over all directions at 20.95°. The Force Levels 5 N and 8 N result in higher correct perception rates. At the medium level, the 15° stimulus exceeds the JND. The values for the spatial and normal force reach 1.29 N and 4.83 N, respectively, whereas the stimulus corresponding to the average JND would have about 1.06 N and 4.88 N. The amount of correct answers per Tangential Component increases until 25° (up to: 86% up; 86% down; 89% left; 79% right).

At the high Force Level at least 50% correct answers occurred within 10° Tangential Component (tangential force: 1.39 N; normal force: 7.88 N). Here, the stimulus corresponding to JND would consist of 1.25 N tangential force and 7.90 N normal force, considering the average JND of the directions with 9.05°. Again, higher spatial force changes lead to a higher amount of correct answers. 25° Tangential Component evoke correct answers of about 90% in all directions. Table A1 presents detailed results about the correct answers of the subjects. The mean values of correct answers over all subjects per Direction and Tangential Component is given.

The results provide evidence that the minimal angle needed to reliably detect a spatial force, is depending on the total amount of force applied.

**Figure 5:**
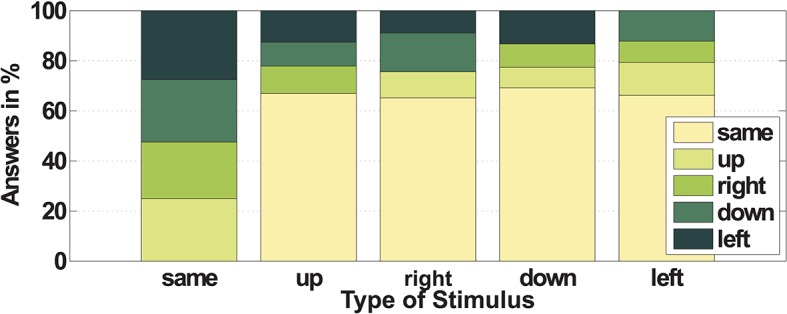
Wrong answers. For each of the commanded directions the perceived wrong decisions *same, up, right, down*, and *left* are depicted in percent (wrong direction was selected by the subjects); each bar illustrates the wrong decision for the respective applied direction.

The factor Direction showed a significant influence, whereas the direction *right* has an influence on the outcome measures. A further analysis, which is illustrated in Figure 5, shows that there is no general confusions in terms of two directions across all subjects. In two-third of wrong decisions, subjects have chosen the answer *same*. The remaining three possible wrong answers are almost equally distributed within each Direction. Each of the directions has been chosen wrongly in 8–15% of the cases.

**Figure 6:**
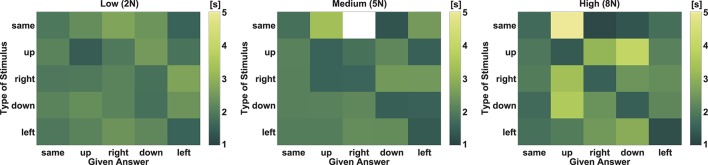
Required time for answering. Illustration of the needed answering time across all subjects per Force Level. Excluded are outliers with times more than 12 s. The y-axes shows the force which was applied to the subjects' toe and the x-axis encodes the Direction which was chosen by the subjects. The diagonal corresponds to the correct answers. The white box illustrates a confusion which did not occur.

Additionally, we examined subjects' decision time, i.e., the time required to take a decision to an individual stimulus. The time recording started with the onset of the stimulus — i.e., the 250 ms needed to apply the full stimulation force is included. The mean time for the decision for all stimuli was about 1.7 s. Hence, a decision time of more than 12 s appears to be unrealistic and therefore was defined as an outlier. According to this, 11 decisions were not considered for the time analysis. These outliers appeared when subjects paused the test session for example to ask questions to the supervisor of the experiment. Focusing on the decision time, the lowest Force Level of 2 N showed a significant difference in comparison to 5 N and 8 N. Figure 6 depicts in form of a confusion matrix per Force Level the average times required for answering. Mean times between 1.3 and 5.5 s occur. The diagonal represents the required reaction time for the correct answers. The white box illustrates a confusion which did not occur. The confusion matrix for the lowest Force Level shows in contrast to the other Force Levels least variation. The decision time required for correct and wrong answers at 2 N is comparable. The other two matrices, especially the one for 8 N, show shorter decision times for the correct answers.

Furthermore, we had a look on effects of learning or fatigue between the three repetitions of the 24 different stimuli and found no considerable variation across these repetitions. For the analysis, the success rate per repetition was used. An overall analysis of learning or fatigue effects in terms of subject's performance, across all subjects and the whole test procedure, cannot be performed because of the different permutations of Force Levels.

### 3.2. Normal test

**Figure 7:**
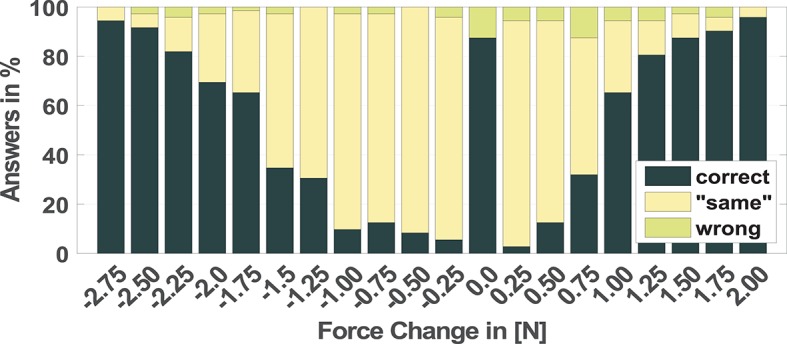
Recorded answers for the NT for all force stimuli between −2.75 N and +2.0 N, while the reference force of 5 N is subtracted. Here 0.0 N corresponds to 5 N with no force change; all negative values correspond to a decreasing stimuli and all positive values correspond to an increasing stimuli; in dark green, correct answers in percent; in yellow, decision to same instead of correct direction; in bright green, decision to the wrong direction.

The results of the NT show that the amount of Force Change (−2.75 N . +2.0 N), the Directions (increase - decrease) as well as the interaction between these factors play a significant role for its discrimination (with increased forces as base: β_*Direction, decrease*_ with std. error = 0.45 *p*-value < 2e-16, β_*Direction, decrease*_ : β_*force*_ with std. error 0.35 and a *p*-value of 5.71e-05). The 20 blue bars in Figure 7 represent the correct responses for the different stimuli in percent. The bar with the label 0.0 refers to *same* during the stimulation phase. The positive values from 0.25 N to 2.0 N describe increasing force stimuli and the negative values represent the decreasing forces. The result shows that correct answers increase with an increase in force. The amount of correct answers at +0.25 N is about 3%. The change of +1.0 N in comparison to the reference force shows 65% and a change of +2.0 N 96% correct answers, respectively. The stimuli with reduced normal force shows as well a higher success rate with higher changes in force. The range between −0.25 N and −1.0 N causes only a correct perception of a maximum of 13% at −0.75 N. The result of the decreased force stimulus of −1.25 N and −1.5 N achieves results comparable to the +0.75 N stimulus. Starting with a force change of −1.75 N (65% correct answers) the amount of correct answers increases continuously with higher changes. The stimulus of −2.75 N produced 95% correct responses. Figure 7 also illustrates the wrong decisions of the subjects during the test. Similar to the TT, in most of the cases of a wrong decision *same* was chosen.

The logistic regression model allowed to estimate a 50%-JND as well. The JND of the NT was at 0.95 N for the increasing force and at −1.62 N decreasing force with the reference force of 5 N.

### 3.3. Secondary results

The questionnaire about mental demand, the self-estimation performance, frustration level, and comfort were rated with respect to the test results. The mental demand and frustration was higher in the level with lower acting force and the performance at the highest Force Level was perceived as the best. However, the subjects felt more comfortable during the application of the lowest Force Level, followed by the medium and the high Force Level.

Additionally a Touch Test based on *von Frey Filaments* has been performed before and after the test cycles of the four main tests. Sensory evaluators were applied to the same effective area as in the main tests. The test allows the identification of the minimal noticeable touch force at the skin. The evaluator size and consequently the acting force were increased until the person could notice at least six out of ten trials. The results are of secondary importance for this paper and hence not mentioned before. Neither the results of the preceding nor of the succeeding test showed any correlation to the Normal or Tangential Tests (*r*-value of about −0.35 and 0.16 *p*-values larger than 0.05).

## 4. Discussion and conclusion

We investigated the ability of 24 subjects to discriminate spatial forces given to their right big toe. We varied force amplitude, relation between tangential and normal component, as well as the acting direction while asking subjects for the perceived changes. The experimental protocol was divided into two tests, namely the Tangential and Normal Test, separating for the influences of direction and amplitude. We found the Degree of Tangential Component, the Force Level and the Direction having a significant influence on subjects' success in perceiving the applied direction. Moreover, we found an influence whether the force amplitude increases or decreases, meaning that subjects were significantly better to sense an increase in force compared to a decrease.

In principal, these results provide evidence for the basic purpose of our study, meaning that subjects were able to discriminate spatial forces at the toe varying in amplitude and Tangential Component. Although subjects were able to recognize all directions, the direction *right* could be perceived significantly worse compared to the other directions. The reason for this effect can not be determined from the result of our study, but it would be of interest to investigate whether this effect can analogously be found on the left big toe.

Our study shows that the directional discrimination threshold at the toe is clearly increased compared to that at the fingertips. Panarese and Edin (2011) identified a minimal tangential discrimination threshold of about 7.1° for the fingertips. The threshold is valid for a total force of 5 N. At the toe, the respective JND is between 11.9° and 12.2° depending on the direction, at the Force Level of 5 N. When applying 8 N to the toe, lower JNDs with about 9° are reached. This is not surprising due to physiological differences between the plantar and the palmar skin (Kennedy and Inglis, 2002). The toe consists of a thicker skin with less mechanoreceptors. Additionally, the distribution of these receptors is different in comparison to the fingers. Moreover, the tactile sensation at the toe is primarily used for balancing during walking and standing. It was a new experience for the subjects to recognize forces at their toe and to assign them to certain directions. The results show that there is a difference in terms of perceiving forces in comparison to the fingers. Nevertheless, a directional discrimination at the toe is possible, but with less accuracy compared to the fingertips.

The applied forces to the toe can describe force feedback during gasping. This type of force feedback represents a natural modality (i.e., force information is fed back as force) at a non-natural stimulation site. The natural stimulation modality offers the advantage that no relearning of the provided stimulus is necessary. Nevertheless, it needs to be investigated, whether the non-natural stimulation location may reduce the acceptance in possible applications. Methods like direct intraneural electrical stimulation or reinnervation techniques can potentially allow for a more natural feedback. However, these invasive techniques are not yet widely available and stimulation to the surface of the skin may provide a viable alternative. Here, the glabrous skin offers a better resolution than hairy regions of the body, when considering the two-point discrimination threshold. With respect to the two-point discrimination threshold, the toe is — besides the skin of the face — the only region of the body that offers values close to those of the hand (Weinstein, 1968). Stimulation of glabrous skin instead of hairy skin seems to be advantageous also from a physiological point of view.

Furthermore, the results indicate that a minimal Tangential Component is needed for reliable (50%-JND) directional discrimination. The medium and highest Force Level reached recognition rates of more then 50% with Tangential Components of more than 1 N. The lowest level does not exceed this force. However, in a friction based setup, a high total force is needed in order to apply a large Tangential Component. While higher Force Levels lead to more clear results, the questionnaire showed that subjects' comfort was significantly reduced. By realizing higher friction between stimulation device and skin, this issue can be compensated for.

**Figure 8:**
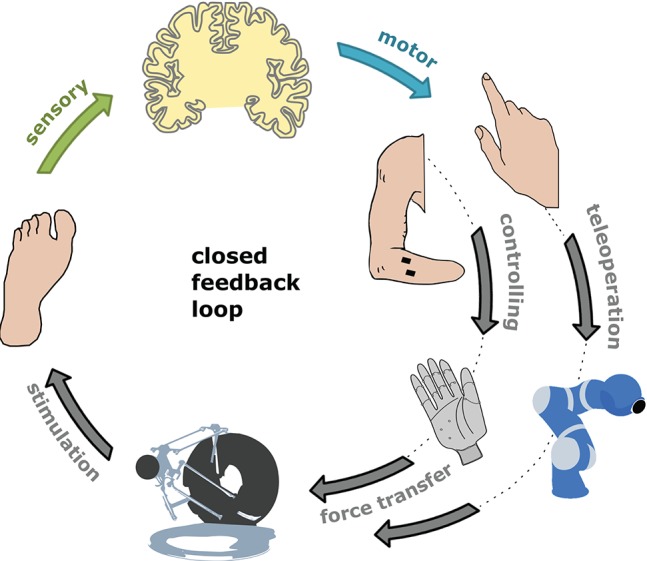
Idea behind. Schematically illustration of the closed feedback-loop by feeding back force information to the human's toe. Two scenarios are considered: controlling a prosthesis and the teleoperation of a robotic device.

In a previous proof of concept we could show that force feedback to the toe can be integrated into the sensorimotor-control, when teleoperating a robotic arm in a force task (Hagengruber et al., 2017). Subjects teleoperated blind-folded a DLR Light-Weight Robot by external optical tracking of their index finger. The task was to push a toy train along the rails. The only feedback of the performed task was presented as force feedback to the subjects' toe. The stimulation of the toe was comparable to this work, despite that the force was applied continuously and presented the forces which were measured at the robots end-effector. With this earlier result and the results obtained in this work, we assume that at least from the physiological point of view, force feedback to the toe can be used for applications in telepresence scenarios or prosthetics. A schematically illustration of such applications can be seen in Figure 8. A practicable technical application is not existent yet. However, using the findings of this work, it is possible to determine a mapping function, to ensure that the tactile stimuli provided to the toe can actually be perceived by the subject. Provided with force information to the toe, users may be able to improve control of such an assistive device. People who rely on a prosthetic hand could get the possibility for a more precise and natural interaction with their environment. Finally, such devices could help in future to further increase the personal acceptance of assistive technologies by increasing their practicality. Moreover, it would be of interest to see, whether similar results can be obtained from stimulation of the other toes, and finally whether subjects are able to discriminate different force vectors at multiple toes, simultaneously.

## Author contributions

AH developed the device, researched the literature, designed the experiments and statistical model, conducted the experiments, analyzed the data, and wrote the manuscript. HH and JV developed the idea, contributed to the experiment and statistical model design, contributed to the analysis and interpretation of data, and revised the work.

### Conflict of interest statement

The authors declare that the research was conducted in the absence of any commercial or financial relationships that could be construed as a potential conflict of interest.

## Appendix

**Table A1:** Mean values of correct answers of three trials over all subjects per Direction and Tangential Component; Results for **(A)** Level Low; **(B)** Level Medium; **(C)** Level High.

**Change**	**Up**	**Down**	**Left**	**Right**
**(A)**				
	**Low [mean ± std]**			
5°	0 ± 0	0 ± 0	0.04 ± 0.2	0.16 ± 0.63
10°	0.46 ± 0.59	0.54 ± 0.83	0.29 ± 0.63	0.20 ± 0.51
15°	1.08 ± 1.21	1.20 ± 1.41	0.88 ± 1.07	0.96 ± 1.20
20°	1.65 ± 1.24	1.79 ± 1.41	1.54 ± 1.02	1.21 ± 1.18
25°	1.79 ± 1.14	1.95 ± 1.08	1.75 ± 1.15	1.71 ± 1.04
**(B)**				
	**Medium [mean ± std]**			
5°	0.38 ± 0.64	0.16 ± 0.48	0.04 ± 0.2	0.20 ± 0.59
10°	1.37 ± 1.21	1.58 ± 1.14	1.83 ± 1.13	1.54 ± 1.14
15°	2.16 ± 1.09	2.21 ± 1.06	2.21 ± 0.83	1.92 ± 1.05
20°	2.63 ± 0.77	2.45 ± 0.97	2.54 ± 0.83	2.15 ± 1.08
25°	2.58 ± 0.78	2.58 ± 1.02	2.66 ± 0.63	2.38 ± 0.92
**(C)**				
	**High [mean ± std]**			
5°	0.54 ± 0.88	0.38 ± 0.77	0.33 ± 0.63	0.46 ± 0.93
10°	2.25 ± 1.03	2.33 ± 1.09	2.25 ± 0.98	1.79 ± 1.21
15°	2.5 ± 0.78	2.58 ± 0.65	2.71 ± 0.55	2.67 ± 0.56
20°	2.75 ± 0.61	2.71 ± 0.63	2.58 ± 0.88	2.63 ± 0.77
25°	2.75 ± 0.68	2.75 ± 0.53	2.75 ± 0.53	2.66 ± 0.76
